# Expansion of base excision repair compensates for a lack of DNA repair by oxidative dealkylation in budding yeast

**DOI:** 10.1074/jbc.RA119.009813

**Published:** 2019-07-18

**Authors:** Suzanne J. Admiraal, Daniel E. Eyler, Michael R. Baldwin, Emily M. Brines, Christopher T. Lohans, Christopher J. Schofield, Patrick J. O'Brien

**Affiliations:** ‡ Department of Biological Chemistry, University of Michigan Medical School, Ann Arbor, Michigan 48109-0600; § Department of Chemistry, University of Oxford, Oxford OX1 3TA, United Kingdom

**Keywords:** base excision repair (BER), DNA repair, Saccharomyces cerevisiae, translation regulation, DNA damage, hydroxylase, AlkA, AlkB, Mag1, oxidative dealkylation, Tpa1

## Abstract

The Mag1 and Tpa1 proteins from budding yeast (*Saccharomyces cerevisiae*) have both been reported to repair alkylation damage in DNA. Mag1 initiates the base excision repair pathway by removing alkylated bases from DNA, and Tpa1 has been proposed to directly repair alkylated bases as does the prototypical oxidative dealkylase AlkB from *Escherichia coli*. However, we found that *in vivo* repair of methyl methanesulfonate (MMS)-induced alkylation damage in DNA involves Mag1 but not Tpa1. We observed that yeast strains without *tpa1* are no more sensitive to MMS than WT yeast, whereas *mag1*-deficient yeast are ∼500-fold more sensitive to MMS. We therefore investigated the substrate specificity of Mag1 and found that it excises alkylated bases that are known AlkB substrates. In contrast, purified recombinant Tpa1 did not repair these alkylated DNA substrates, but it did exhibit the prolyl hydroxylase activity that has also been ascribed to it. A comparison of several of the kinetic parameters of Mag1 and its *E. coli* homolog AlkA revealed that Mag1 catalyzes base excision from known AlkB substrates with greater efficiency than does AlkA, consistent with an expanded role of yeast Mag1 in repair of alkylation damage. Our results challenge the proposal that Tpa1 directly functions in DNA repair and suggest that Mag1-initiated base excision repair compensates for the absence of oxidative dealkylation of alkylated nucleobases in budding yeast. This expanded role of Mag1, as compared with alkylation repair glycosylases in other organisms, could explain the extreme sensitivity of Mag1-deficient *S. cerevisiae* toward alkylation damage.

## Introduction

**Figure 1.**
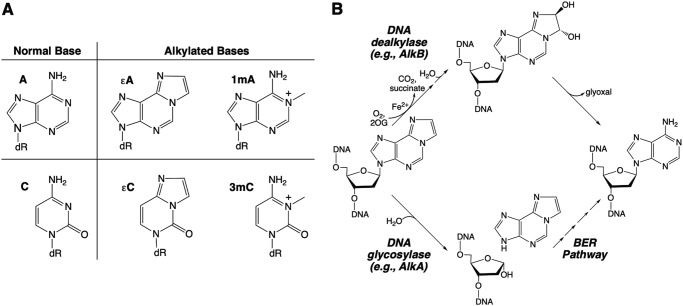
**Alkylation damage to DNA bases and pathways for repair.** *A*, examples of alkylated nucleobases. *B*, ϵA may be repaired by either oxidative dealkylation (*upper* pathway; DRR) or glycosylase-initiated BER (*lower* pathway).

DNA bases can be damaged by alkylation, which may cause mutations that lead to cell death or disease. Several examples of alkylated bases that arise in DNA from endogenous and exogenous sources are shown in Fig. 1*A*. Pathways for repair of alkylated bases in many organisms, including *Escherichia coli* and humans, include base excision repair (BER)^4^ and direct reversal repair (DRR). For example, repair of the alkylated base 1,*N*^6^-ethenoadenine (ϵA) in *E. coli* can be initiated by the DNA glycosylase AlkA and completed by other enzymes in the BER pathway, or DRR can be catalyzed by the DNA dealkylase AlkB in a reaction requiring ferrous iron, oxygen, and 2-oxoglutarate (2OG) (Fig. 1*B*). Other alkylated bases are typically repaired by either BER or DRR. Interestingly, it has been suggested that some bacteria and archaea that appear to lack an AlkB-family oxidative dealkylase instead use BER with an expanded substrate repertoire to repair alkylation damage (1–3). In support of this idea, DNA glycosylases from these organisms have been shown to excise alkylated bases that have more commonly been thought to be substrates for direct repair by AlkB and related DNA dealkylases.

*Saccharomyces cerevisiae* is an important organism for the study of DNA repair, yet the distribution of its alkylation repair between BER and DRR is undefined. Mag1, a DNA glycosylase that is a known homolog of *E. coli* AlkA, is present in *S. cerevisiae* (4, 5). Both Mag1 and AlkA remove a variety of alkylated bases from DNA, and the resultant abasic DNA is then processed by downstream enzymes of the BER pathway. Mag1 is the only DNA glycosylase in *S. cerevisiae* that is known to excise alkylated bases. *S. cerevisiae* also expresses Tpa1, which, like *E. coli* AlkB and many other proteins, is a member of the Fe(II)- and 2OG-dependent dioxygenase superfamily (6). Tpa1 has recently been reported to be a functional homolog of AlkB, acting as an oxidative dealkylase to repair alkylated DNA (7). However, Tpa1 is more closely related to Fe(II)/2OG-dependent dioxygenases that catalyze post-translational hydroxylation of proteins (8–10), and it has been reported to catalyze prolyl hydroxylation of ribosomal protein S23 and to regulate translation (11, 12). We thus set out to clarify the roles of Mag1 and Tpa1 in the repair of alkylation damage to DNA in *S. cerevisiae*.

## Results

### Deletion of tpa1 in yeast does not confer sensitivity to MMS

**Figure 2.**
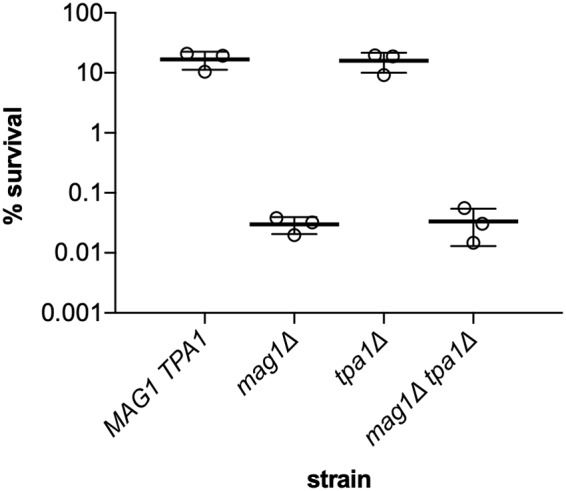
**Cell killing induced by exposure of yeast strains to MMS.** Exponentially growing yeast cells were exposed to 0.3% MMS (v/v) for 1 h, then plated on YPD, and evaluated for survival relative to untreated cells. *Error bars* represent S.D.

The alkylating agent methyl methanesulfonate (MMS) is widely used to generate DNA damage to investigate DNA repair pathways. MMS methylates DNA primarily at the N-7 position of G (7mG), the N-3 and N-1 positions of A (3mA and 1mA), and the N-3 position of C (3mC) (13). We constructed yeast strains lacking *mag1*, *tpa1*, or both genes using standard methods. These strains were exposed to MMS to compare the contributions of Mag1 (the yeast homolog of *E. coli* AlkA) and Tpa1 (a possible functional homolog of *E. coli* AlkB) to the repair of alkylation damage. The results reveal that the *mag1*Δ strain is ∼500-fold more sensitive to 0.3% MMS after 1-h exposure than is WT (Fig. 2), as has been observed previously (14). This is consistent with the established ability of Mag1 to remove alkylated bases, including 7mG and 3mA, from DNA (15). However, the *tpa1*Δ strain and WT are similarly sensitive to MMS, and the *mag1*Δ *tpa1*Δ double-deletion strain is no more sensitive to MMS than is the *mag1*Δ deletion strain alone (Fig. 2).

Our results with *mag1*Δ, but not with *tpa1*Δ, agree qualitatively with previous observations in *E. coli* where both the *alkA* and *alkB* genes were initially discovered in screens for mutants with increased sensitivity to MMS (16, 17). In addition, the MMS sensitivity of an *alkA*Δ *alkB*Δ double-deletion strain was approximately at the level predicted from the additive sensitivity of each of the single mutants (18). Thus, our results validate the importance of Mag1, but not Tpa1, for repair of DNA alkylation damage in *S. cerevisiae*. No members of the Fe(II)/2OG-dependent dioxygenase superfamily other than Tpa1 have been identified as possible AlkB family oxidative dealkylases in *S. cerevisiae* (19, 20). This raises the question of how lesions typically repaired by the DRR protein AlkB in *E. coli*, such as 1mA and 3mC, are repaired in yeast.

### Yeast Mag1 excises alkylated bases that are known substrates of E. coli AlkB

**Figure 3.**
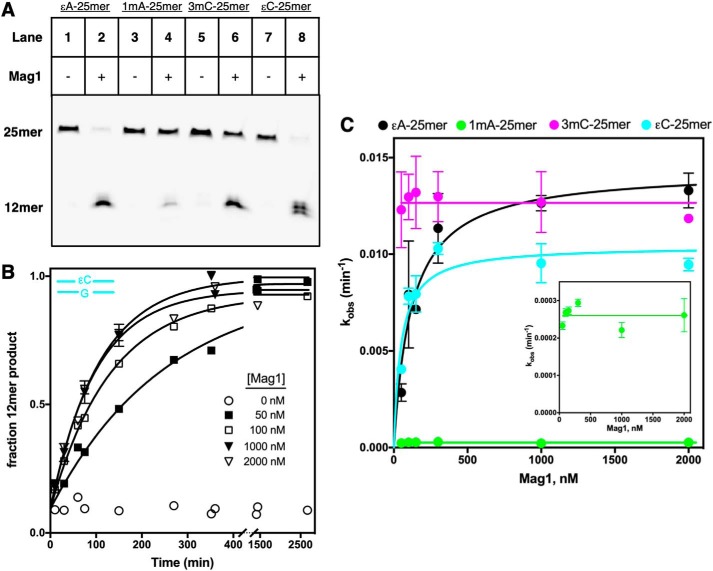
**Mag1 excises alkylated nucleobases that are known substrates for DRR by AlkB.** *A*, Mag1 excises ϵA, 1mA, 3mC, and ϵC from duplex DNA. After 24-h incubation of 25mer DNA duplexes (Table S1) containing the indicated central bp with Mag1, the abasic sites were cleaved with sodium hydroxide, and the samples were analyzed on a 17.5% denaturing polyacrylamide gel. Control reactions (*lanes 1*, *3*, *5*, and *7*) show that the oligonucleotides remain intact in the absence of Mag1. The two product bands in *lane 8* result from the milder hydrolysis conditions used to cleave abasic sites in reactions containing ϵC and correspond to species with 3′-phosphate or 3′-deoxyribose phosphate termini, which migrate slightly differently (see “Experimental procedures”). *B*, representative time courses for single-turnover excision of ϵC from 5 nm ϵC-25mer by varying concentrations of Mag1. The data were fit by a single exponential. The average of duplicate reactions is shown, and *error bars* represent S.D. *C*, dependence of the single-turnover rate constant for alkylated base excision from ϵA-25mer, 1mA-25mer, 3mC-25mer, and ϵC-25mer substrates on the Mag1 concentration. Hyperbolic dependence of the single-turnover rate constant on the Mag1 concentration was observed for ϵA-25mer and ϵC-25mer with *k*_max_ of 0.014 ± 0.001 min^−1^ and a *K*_1/2_ value of 120 ± 30 nm for ϵA-25mer and *k*_max_ of 0.010 ± 0.001 min^−1^ and a *K*_1/2_ value of 48 ± 18 nm for ϵC-25mer. *K*_1/2_ values were too low to be measured for 1mA-25mer and 3mC-25mer, but *k*_max_ of 0.013 ± 0.001 min^−1^ for 3mC-25mer and *k*_max_ of 0.00026 ± 0.00003 min^−1^ for 1mA-25mer were determined by averaging all measured *k*_obs_ values for each substrate. The *inset* shows a rescaled plot of the 1mA-25mer data. The average of duplicate reactions is shown, and *error bars* represent S.D.

We considered the possibility that yeast, like some bacteria and archaea, utilize DNA glycosylases to remove alkylated bases that are more commonly considered to be substrates for DRR by AlkB and related DNA dealkylases (1–3). To test the idea that the DNA glycosylase Mag1 may be playing this role in yeast, we surveyed its activity toward a group of known AlkB substrates, including 1mA and 3mC (Fig. 1*A*) (21–24). The ϵA lesion is a known substrate of Mag1 (25); we used a gel-based assay to show that the other alkylated bases shown in Fig. 1*A* are also substrates of Mag1 when embedded in 25mer duplex DNA oligonucleotides (Table S1 and Fig. 3*A*). Single-turnover kinetic measurements ([Mag1] > [substrate]) for each substrate showed that Mag1 has much higher *k*_max_ values for excision of 3mC, ϵC, and ϵA than for excision of 1mA (Figs. 3 and S1). Furthermore, although *k*_max_ values for excision of 3mC, ϵC, and ϵA are very similar (0.010–0.014 min^−1^; Fig. 3*C* and Table 1), we were only able to measure an upper limit for *K*_1/2_ for 3mC, suggesting that it binds to Mag1 very tightly and is a preferred substrate. We were also only able to measure an upper limit for *K*_1/2_ for 1mA, suggesting that it, like 3mC, binds to Mag1 tightly, despite its comparatively low *k*_max_ value of 0.00026 min^−1^ (Table 1). Excision of these alkylated bases by Mag1 did not occur when they were present in 25mer ssDNA oligonucleotides (Fig. S2).

**Table 1:** **Kinetic parameters for excision of alkylated bases from dsDNA by Mag1 and AlkA**

	Mag1	AlkA	Relative Mag1/AlkA
**ϵA-25mer**			
*k*_max_ (min^−1^)*^a^*	0.014 ± 0.001	0.0058 ± 0.0001	2.4
*K*_1/2_ (nm)*^a^*	120 ± 30	780 ± 40	0.15
*k*_max_/*K*_1/2_ (m^−1^ min^−1^)*^a^*	120,000 ± 30,000	7,400 ± 400	16
Relative *k*_cat_/*K_m_^b^*	0.98 ± 0.13	1.3 ± 0.4	
**3mC-25mer**			
*k*_max_ (min^−1^)*^a^*	0.013 ± 0.001	0.0023 ± 0.0001	5.7
*K*_1/2_ (nm)*^a^*	<10*^c^*	540 ± 10	<0.02
*k*_max_/*K*_1/2_ (m^−1^ min^−1^)*^a^*	>1,300,000	4,300 ± 200	>300
Relative *k*_cat_/*K_m_^b^*	>60*^d^*	0.79 ± 0.18	
**ϵC-25mer**			
*k*_max_ (min^−1^)*^a^*	0.010 ± 0.001	No reaction	
*K*_1/2_ (nm)*^a^*	48 ± 18	No reaction	
*k*_max_/*K*_1/2_ (m^−1^ min^−1^)*^a^*	210,000 ± 80,000	<12*^e^*	>18,000
Relative *k*_cat_/*K_m_^b^*	2.3 ± 0.5	<0.1*^f^*	
**1mA-25mer**			
*k*_max_ (min^−1^)*^a^*	0.00026 ± 0.00003	0.0012 ± 0.0001	0.22
*K*_1/2_ (nm)*^a^*	<10*^c^*	3,400 ± 600	<0.0029
*k*_max_/*K*_1/2_ (m^−1^ min^−1^)*^a^*	>26,000	350 ± 70	>74
Relative *k*_cat_/*K_m_^b^*	0.70 ± 0.10	0.21 ± 0.05	

*^a^* At 25 °C and pH 7.5 (50 mm MOPS, 0.2 m KOAc, 1 mm EDTA, 1 mm TCEP, 0.1 mg/ml BSA).

*^b^* From competition experiments with ϵA-19mer at 25 °C and pH 7.5 (50 mm MOPS, 0.1 m NaCl, 1 mm EDTA, 1 mm TCEP, 0.1 mg/ml BSA).

*^c^* The single-turnover rate constants for base excision from 3mC-25mer or 1mA-25mer were independent of Mag1 concentration at 50 nm Mag1, the lowest concentration used in our reactions, and we estimate that we would have been able to detect a dependence for *K*_1/2_ of 10 nm or higher (Fig. 3*C*).

*^d^* See Fig. S3B for calculation of limit.

*^e^* See Fig. S6D for calculation of limit.

*^f^* See Fig. S7D for calculation of limit.

**Figure 4.**
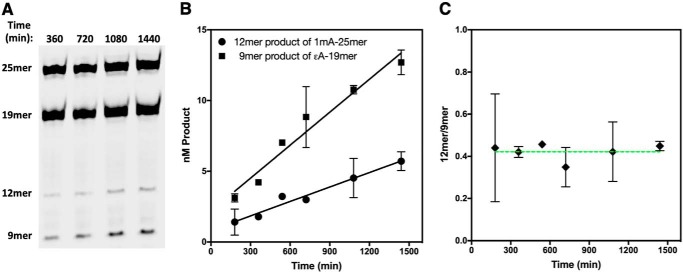
**Representative data for determination of Mag1 relative *k*_cat_/*K_m_* values.** *A*, a typical denaturing polyacrylamide gel showing a time course for Mag1-catalyzed glycosylase activity toward a mixture of 1mA-25mer and the reference substrate, ϵA-19mer (Table S1). Cleavage of 1mA-25mer gives a labeled 12mer product, and cleavage of the reference substrate gives a labeled 9mer product. Mag1 was 50 nm, 1mA-25mer was 300 nm, and ϵA-19mer was 500 nm. *B*, quantitation of time points shown in *A* and identical reactions. The average of duplicate reactions is shown, and *error bars* represent S.D. The relative *k*_cat_/*K_m_* value of 0.70 ± 0.10 for 1mA-25mer with respect to ϵA-19mer was determined from the linear initial rates and the initial substrate concentrations (see “Experimental procedures”). *C*, at any given time point in *B*, the ratio of the two products is the same within error.

Although our single-turnover measurements reveal that Mag1 excises alkylated bases from known AlkB substrates, comparison of *k*_cat_/*K_m_* values for different substrates provides a better measure of substrate specificity. These apparent second-order rate constants, commonly referred to as specificity constants, report on all of the steps up to and including the first irreversible step (26); for Mag1 they include the substrate binding and *N*-glycosidic bond hydrolysis steps. We used direct competition assays to determine relative *k*_cat_/*K_m_* values for each Mag1 25mer substrate with reference to ϵA-19mer (Figs. 4 and S3). Compared with the previously reported Mag1 substrate, ϵA, both 3mC and ϵC have larger relative *k*_cat_/*K_m_* values, and 1mA has a relative *k*_cat_/*K_m_* value within 2-fold of the relative *k*_cat_/*K_m_* value for ϵA (Table 1). Indeed, 3mC was excised so much faster than ϵA in our competition reactions that we were unable to detect the reaction of the ϵA-19mer reference substrate until most of the 3mC-25mer had undergone reaction. Therefore, only a lower limit of relative *k*_cat_/*K_m_* >60 for 3mC-25mer could be obtained, indicating that Mag1 has at least a 60-fold preference for 3mC over ϵA. These results indicate that 3mC is a substantially better substrate for Mag1 than ϵA, ϵC, or 1mA, which all have similar relative *k*_cat_/*K_m_* values of 0.70–2.3 (Table 1).

### Purified Tpa1 hydroxylates proline but does not oxidatively dealkylate DNA

**Figure 5.**
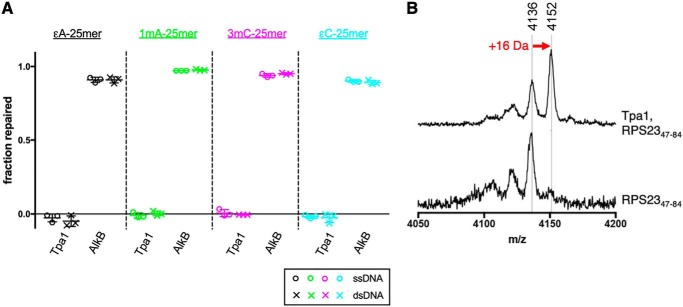
**Tpa1 and AlkB are not functionally homologous.** *A*, AlkB repairs both ss and ds 25mers containing a central ϵA, 1mA, 3mC, or ϵC, but no DNA repair activity is observed for Tpa1 using the same substrates and reaction conditions. Reactions contained 5 μm AlkB or Tpa1, 100 nm ss or ds 25mer DNA, and appropriate Fe(II) cofactor and 2OG cosubstrate and were incubated at 37 °C for 1 h. Reactions were performed in triplicate. *B*, MALDI-TOF MS analyses of 25 μm RPS23_47–84_ peptide (calculated mass, 4136 Da) with or without 2 μm Tpa1 and appropriate Fe(II) cofactor and 2OG cosubstrate at room temperature for 10 min (see “Experimental procedures” for details). The mass increase of 16 Da corresponds to hydroxylation of the peptide by Tpa1.

Following our finding that the *tpa1*Δ yeast strain showed no MMS sensitivity beyond that of WT *S. cerevisiae*, we tested the ability of purified Tpa1 to repair alkylated ssDNA and duplex DNA *in vitro*. Recombinant Tpa1 was produced in *E. coli*, purified, and assayed alongside purified AlkB in the presence of Fe(II) and 2OG. Whereas AlkB completely repaired ϵA, 1mA, 3mC, and ϵC embedded in either ssDNA or dsDNA within 1 h, Tpa1 showed no repair activity on any of these eight substrates even after 29 h (Figs. 5*A* and S4).

We analyzed AlkB, Tpa1, and closely related members of the Fe(II)/2OG-dependent dioxygenase superfamily from human, fruit fly, and fission yeast using the Conserved Domain Database from the NCBI (27) (Table S2 and Fig. S5). Notably, N-terminal prolyl hydroxylase domains (P4Hc and EGL9) are conserved in Tpa1 and its close homologs, which extend to humans, consistent with crystallographic studies (8–10). A different conserved dioxygenase domain is associated with AlkB and its close homologs (6). In addition, Tpa1 and its close homologs contain a distinctive C-terminal dioxygenase domain (Ofd1-CTDD) that is missing the H*X*(D/E)…H Fe(II)-binding triad and hence is unlikely to have oxygenase activity (8–10). These observations are consistent with previous studies concluding that Tpa1 is more closely related to Fe(II)/2OG-dependent dioxygenases that catalyze post-translational hydroxylation of proline than to dioxygenases like AlkB that modify nucleic acid substrates (8–10).

We next tested our purified Tpa1 for its reported prolyl hydroxylase activity (11). Hydroxylation of a 38-residue fragment of human ribosomal protein RPS23 was detected as a 16-Da increase in the mass of the peptide after a 10-min incubation with Tpa1 (Fig. 5*B*). The RPS23 peptide contains Pro-62, the residue determined to be hydroxylated by OGFOD1, the human homolog of yeast Tpa1, in previous studies (28).

### Comparison of activity of yeast Mag1 and E. coli AlkA toward known substrates of E. coli AlkB

AlkA is a DNA glycosylase from *E. coli* that is a homolog of Mag1 (4, 5). Because both AlkA (BER) and AlkB (DRR) are present in *E. coli*, whereas only Mag1 appears to be present in *S. cerevisiae*, we wondered whether AlkA and Mag1 would react differently with known AlkB substrates. We therefore performed single-turnover (Fig. S6) and competition assays (Fig. S7) with AlkA, as described above for Mag1, to compare the preferences of the two glycosylases for the known AlkB substrates in Fig. 1*A*.

We confirmed that *E. coli* AlkA excises ϵA from dsDNA (Fig. S6*A*) as has been reported previously (25). We also observed slow AlkA-catalyzed excision of 1mA from 1mA-25mer (Fig. S6*B*) and of 3mC from 3mC-25mer (Fig. S6*C*), although excision of these bases from dsDNA by AlkA was not detected in previous studies (1, 29). No excision of ϵC from ϵC-25mer by AlkA could be detected (Fig. S6*D*), so only limits for its *k*_max_/*K*_1/2_ and relative *k*_cat_/*K_m_* values are reported in Table 1.

Overall, the single-turnover kinetic parameters *k*_max_, *K*_1/2_, and *k*_max_/*K*_1/2_ are more favorable for Mag1 than for AlkA for all substrates tested (Table 1). For excision of ϵA, for example, Mag1 has ∼2-fold larger *k*_max_ and ∼7-fold lower *K*_1/2_ values that correspond to a 16-fold larger *k*_max_/*K*_1/2_ value for Mag1 than for AlkA with this established substrate. However, this Mag1/AlkA ratio of 16 for excision of ϵA is dwarfed by the limits of >300, >18,000, and >74 that we obtained for the *k*_max_/*K*_1/2_ ratios of the two glycosylases for excision of 3mC, ϵC, and 1mA, respectively (Table 1). These large ratios indicate that 3mC, ϵC, and 1mA in dsDNA are all much better substrates for Mag1 than for AlkA and suggest that Mag1 may be important for their repair in yeast.

The relative *k*_cat_/*K_m_* values for excision of 3mC, ϵC, and 1mA by AlkA, obtained from competition experiments, are all less than 1, indicating that they are inferior substrates relative to the well-characterized AlkA substrate ϵA (Fig. S7 and Table 1). In contrast, for Mag1 the relative *k*_cat_/*K_m_* values are similar to or greater than 1 for excision of 3mC, ϵC, and 1mA, indicating that these bases are similar to or preferred over ϵA as substrates (Table 1). This result is consistent with the proposal that Mag1, by engaging with known AlkB substrates, may compensate for the absence of an AlkB-family oxidative dealkylase in *S. cerevisiae*.

## Discussion

Yeast missing *mag1* are ∼500-fold more susceptible to cell death than WT yeast after exposure to the alkylating agent MMS, but yeast missing *tpa1* do not show increased susceptibility (Fig. 2). This led us to question the proposed direct participation of Tpa1 in repair of alkylated DNA (7) and to hypothesize that Mag1 may play a larger role in this process than has been appreciated. We showed that Mag1 excises alkylated bases present in duplex DNA that are known to be substrates of the DRR enzyme AlkB, including 3mC and 1mA. Although Tpa1, like AlkB, is a member of the Fe(II)/2OG-dependent dioxygenase superfamily, Tpa1 does not repair any of the AlkB substrates we tested in either ssDNA or duplex DNA. Instead, we confirmed that Tpa1 is a prolyl hydroxylase, consistent with its close sequence and structural relatedness to other prolyl hydroxylases and its role in regulation of translation (Table S2, Fig. S5, and Refs. 8–12). We conclude that Tpa1 is not a DNA dealkylase and that yeast fare without one, due at least in part to the expanded substrate repertoire of the DNA glycosylase Mag1.

Other organisms that appear to lack an AlkB-family oxidative dealkylase and have been proposed to instead use DNA glycosylase-initiated BER to accomplish repair are *Deinococcus radiodurans*, *Archaeoglobus fulgidus*, and *Bacillus cereus* (1–3). Like yeast, each of these organisms has a glycosylase that excises 3mC and 1mA from DNA. From a physiological perspective, it may be beneficial for organisms that can grow in the absence of oxygen, such as yeast and *B. cereus* (30), or for organisms that are obligate anaerobes, such as the methanoarchaea *A. fulgidus* (31), to avoid reliance on AlkB-like dioxygenases for DNA repair (20, 32). However, *D. radiodurans* is an obligate aerobe (33). In addition to BER, it is possible that novel repair mechanisms, nucleotide excision repair, or deployment of translesion DNA polymerases may replace DNA dealkylation in some organisms.

Mag1 compensates for the absence of an AlkB-family dealkylase in yeast by excising 1mA and 3mC, which are important substrates of AlkB (21, 22). Although AlkB is able to repair ϵC (23), the primary enzyme involved in repair of ϵC in *E. coli* is the DNA glycosylase MUG (34, 35). *S. cerevisiae* lacks a MUG homolog (36), but we have shown that ϵC is a substrate for Mag1. Notably, ϵC is not a substrate for AlkA, the homolog of Mag1 from *E. coli*. Thus, in addition to compensating for the absence of an AlkB homolog, Mag1 may also compensate for the absence of a MUG homolog in yeast.

The canonical AlkB substrates 3mC and 1mA are both excised by Mag1, and their *K*_1/2_ values of <10 nm indicate that DNA containing either damaged base binds to Mag1 very tightly. However, turnover of 1mA by Mag1 is slow, with a half-time of days. This modest activity raises the possibility that Mag1 may recognize and bind to some alkylated bases but not ultimately excise them. According to this model, the Mag1·DNA complex would serve as a damage sensor to recruit components of nucleotide excision repair or other repair pathways that would then carry out repair.

Our studies of Mag1 fit in with a general theme of DNA repair: organisms use different combinations of repair proteins and pathways to remove identical damage. It is common for repair proteins to have overlapping substrate specificities, which leads to redundancy in DNA repair in some organisms. Apparently, gain or loss of redundancy and other shifts in the overall balance of DNA repair have occurred throughout evolution. Selective pressure based upon the amounts and types of DNA damage to which an organism is exposed due to its specific physiology and environment could drive such changes. Given the potentially lethal consequences of DNA damage to genomes, such flexible and continually evolving DNA repair strategies are vital.

## Experimental procedures

### Construction of yeast strains

BY4741, *mag1*Δ*::kanMX*, and *tpa1*Δ*::kanMX* strains were obtained from the yeast knockout collection (37). Derivative *URA3*^+^ strains were constructed and verified using standard methods (38) and are listed in Table S3. The WT *URA3* sequence was amplified by PCR using genomic DNA from yeast strain YW151 (see Table S4 for primers) and transformed into each of the three strains by the lithium acetate procedure (39). Integrants were selected on media lacking uracil and tested for sensitivity to 5-fluoroorotic acid. Correct integration was verified by PCR using primers listed in Table S4 (Table S5 and Fig. S8). To construct the *mag1*Δ*::LEU2 tpa1*Δ*::kanMX* double knockout, the *LEU2* cassette was amplified from pRS425 by PCR using the primers in Table S4 and transformed into the *tpa1*Δ*::kanMX* strain. Integrants were selected on media lacking leucine. Correct integration was verified by PCR using primers listed in Table S4 (Table S5 and Fig. S9).

### Analysis of MMS sensitivity of yeast strains

Yeast cultures were inoculated to an optical density of 0.1 at 600 nm (OD_600_) from overnight cultures and grown in yeast extract/peptone/dextrose (YPD) medium to OD_600_ = 0.6. The culture was diluted to OD_600_ = 0.2 with fresh YPD. This cell suspension was further diluted to OD_600_ = 0.1 with YPD (for −MMS control cultures) or 0.6% MMS (v/v) in YPD (for +0.3% MMS cultures). Cell suspensions were incubated at room temperature with rotation for 1 h before diluted aliquots were spread on YPD plates. Colonies were counted after 3–5 days at 30 °C to determine cell survival.

### Purification of proteins

*S. cerevisiae* Mag1 (40) and *E. coli* AlkA (41) were expressed in *E. coli* and purified as described previously. The *S. cerevisiae* gene for Tpa1 was amplified by PCR using BY4741 genomic DNA as a template, cloned into the BamHI and XhoI sites of ppSUMO, and expressed in Rosetta2 *E. coli* (Novagen). Cells were grown overnight in LB broth to inoculate 500-ml terrific broth cultures. These cultures were grown at 37 °C until OD_600_ reached 1.0, and then the temperature was reduced to 18 °C for 1 h prior to induction with 200 μm isopropyl 1-thio-β-d-galactopyranoside. Cells were harvested by centrifugation, and each pellet was resuspended in 100 ml of lysis buffer (25 mm sodium phosphate, 500 mm sodium chloride, 10% (v/v) glycerol, pH 7.4). Cells were lysed by two passes through an Emulsiflex C3 homogenizer (Avestin, Inc.) at 18,000 p.s.i. gauge and centrifuged to clarify. The supernatant was stirred at 4 °C while polyethyleneimine was added to 0.05% (w/v), and then centrifugation was repeated. The protein was bound to a nickel-charged HisTrap HP column (GE Healthcare) and eluted over an imidazole gradient (15–400 mm). Peak fractions were pooled, and the fusion protein was digested by incubation with ULP1 overnight. The protein was then bound to a HiTrap Q HP column (GE Healthcare) and eluted over a sodium chloride gradient. Peak fractions were pooled and concentrated using a centrifugal ultrafiltration cell (Amicon) to a final concentration of 188 μm as determined by the absorbance at 280 nm using the predicted extinction coefficient of 98,780 m^−1^ cm^−1^.

A pET19-derived expression construct for *E. coli* AlkB (42) was transformed into BL21(DE3) *E. coli*, and protein was expressed via autoinduction in 500 ml of terrific broth cultures at 18 °C (43). Cells were harvested via centrifugation and resuspended in 100 ml of lysis buffer (25 mm potassium phosphate, 300 mm sodium chloride, 5% (v/v) glycerol, 5 mm 2-mercaptoethanol, pH 7.4) per pellet. Lysis and nickel-affinity chromatography were performed as described for Tpa1. The tag was removed by incubation with PreScission protease for 1 h. The protein was then bound to a HiTrap SP HP column (GE Healthcare) and eluted over a salt gradient. Peak fractions were concentrated via centrifugal ultrafiltration and then loaded onto a Superdex 200 column equilibrated with storage buffer (20 mm Tris, 100 mm sodium chloride, 0.1 mm TCEP, pH 8.0). Protein was concentrated to 650 μm as determined by the absorbance at 280 nm using the predicted extinction coefficient of 32,430 m^−1^ cm^−1^. An SDS-polyacrylamide gel of the purified proteins used in this study is shown in Fig. S10.

### Synthesis and purification of oligonucleotides

DNA substrates were synthesized by Integrated DNA Technologies, Inc. or the Keck facility at Yale and were purified using denaturing PAGE as described previously (44). Sequences are provided in Table S1. Single-strand DNA concentrations were determined from the absorbance at 260 nm using calculated extinction coefficients. For oligonucleotides containing ϵA, the extinction coefficient was calculated for the same sequence with an A in place of ϵA and corrected by subtracting 9400 m^−1^ cm^−1^ to account for the weaker absorbance of ϵA (45). For oligonucleotides containing 1mA, 3mC, and ϵC, the extinction coefficient was calculated for the same sequence with A in place of 1mA or C in place of 3mC and ϵC. Duplexes were annealed with 1.5-fold excess unlabeled complement.

### Single-turnover glycosylase assays for Mag1 and AlkA

Reactions were carried out at 25 °C in 50 mm MOPS, pH 7.5, 200 mm KOAc, 0.1 mg/ml BSA, 1 mm EDTA, 1 mm TCEP with enzyme in excess of substrate. Typical reactions contained 5 nm fluorescein-labeled DNA and 50 nm–3 μm enzyme (Mag1 or AlkA). Reactions were initiated by adding a small volume of DNA substrate to the remaining reaction components in a final volume of 20–40 μl. Aliquots were withdrawn at various times and quenched with sodium hydroxide to give a final concentration of 0.2 m. Abasic sites were converted into single-strand breaks for most quenched samples by heating at 70 °C for 10 min; ϵC degrades under the standard hydrolysis conditions, so samples containing ϵC were subjected to milder hydrolysis at room temperature for 1 h. The milder hydrolysis gives a mixture of two products, the first from β-elimination on the 3′ side of the abasic site to give an oligonucleotide terminated by an α,β-unsaturated aldehyde fragment and the second from δ-elimination of the aldehyde fragment remaining on the first (46). Only the second product is stable when the standard hydrolysis conditions (70 °C) are used. Hydrolyzed samples were mixed with an equal volume of formamide/EDTA loading buffer and analyzed by PAGE on 17.5% (w/v) gels containing 6.6 m urea. Gels were scanned using a Typhoon imager (GE Healthcare), and emission was measured with a 520BP40 filter following excitation of the fluorescein label at 488 nm. Fluorescence intensities of gel bands were quantified using ImageQuant TL (GE Healthcare) and corrected for the amount of background signal. The data were converted to fraction product (P) and then fit by the single exponential shown in Equation 1. The single exponential included a zero point, which is the fraction of prehydrolyzed substrate in control reactions without enzyme, and an end point term, which is the total fraction of each DNA substrate that could be converted to abasic DNA by Mag1 and AlkA and subsequently hydrolyzed. Different DNA substrates had different end points, which were confirmed in reactions with other glycosylases (data not shown). End points for both ϵA-25mer and ϵC-25mer were 0.95, whereas end points for the more synthetically challenging 1mA-25mer and 3mC-25mer substrates were 0.60 (3, 47).

(Eq. 1) $$\text{Fraction}\;\text{P}=\text{End}\;\text{point}(1-\exp\left(-k_{\text{obs}}t\right)+\text{Zero}\;\text{point}$$

### Competition assays to determine relative k_cat_/K_m_ values for Mag1 and AlkA

Relative specificity for Mag1 and AlkA substrates was determined by competition assays (48) with the substrate ϵA-19mer used as a reference (Table S1). Each substrate was mixed with the reference substrate at varying ratios for total DNA concentrations of 0.8–1.8 μm. Reactions were carried out at 25 °C in 50 mm MOPS, pH 7.5, 100 mm NaCl, 0.1 mg/ml BSA, 1 mm EDTA, 1 mm TCEP. Mag1 or AlkA was added to a final concentration of 50 nm, and the glycosylase activity was followed up to 10% depletion of either substrate. Samples were quenched, hydrolyzed, and analyzed by PAGE as described above. Initial velocities are proportional to the relative *k*_cat_/*K_m_* values as described in Equation 2 (26).

(Eq. 2) $$V_{A}/V_{B}=\left(\left[A\right]\times {\left(k_{\text{cat}}/K_{m}\right)}_{A}\right)/\left(\left[B\right]\times {\left(k_{\text{cat}}/K_{m}\right)}_{B}\right)$$

### Activity assays for Tpa1 and AlkB

DNA repair activity of Tpa1 and AlkB was assessed by incubating 5 μm each enzyme with 100 nm ss or duplex versions of the 25mer DNA substrates shown in Table S1. Reactions were carried out at 37 °C in a 10–20-μl reaction mixture containing 50 mm HEPES pH 7.5, 150 mm NaCl, 0.1 mg/ml BSA, 1 mm TCEP, 2 mm sodium l-ascorbate, 1 mm 2OG, 50 μm ammonium iron (II) sulfate. Repair reactions were quenched with 10 mm EDTA in a solution that contained a 10-fold excess of Mag1 or *Bacillus subtilis* AAG (49) over the DNA substrate. When ssDNA substrates were used in the reaction, an excess of the complementary strand was also included in the quench solution. Quenched samples were incubated at room temperature overnight to allow the DNA glycosylase present to convert any unrepaired DNA into abasic DNA (Mag1 and *B. subtilis* AAG are active in EDTA). Sodium hydroxide was then added to a final concentration of 0.2 m, samples were heated at 70 °C for 10 min to convert abasic sites into single-strand breaks, and the hydrolyzed samples were analyzed by denaturing PAGE on 20% (w/v) gels as described above. This glycosylase treatment converts unrepaired 25mer DNA into 12mer DNA, but 25mers that have been repaired by AlkB or Tpa1 remain intact and appear as slower migrating bands on gels (50). *B. subtilis* AAG was used instead of Mag1 to treat 1mA-containing DNA samples because of its faster kinetics with this substrate under these conditions (data not shown). Mock DNA repair reactions were performed for each DNA substrate in the absence of Tpa1 or AlkB as a positive control for the DNA glycosylase activity of Mag1 and *B. subtilis* AAG and to reveal the maximum amount of repairable DNA in each initial sample. As noted above, end points for both ϵA-25mer and ϵC-25mer were 0.95, whereas end points for 1mA-25mer and 3mC-25mer substrates were 0.60 (3, 47).

The hydroxylation activity of Tpa1 was assessed by incubating 2 μm enzyme with 25 μm RPS23_47–84_, a 38-amino acid fragment of human ribosomal protein RPS23 (sequence, AKGIV LEKVG VEAKQ PNSAI RKCVR VQLIK NGKKI TAF-CONH_2_). Reaction mixtures contained 50 mm HEPES, pH 7.5, 100 μm sodium l-ascorbate, 20 μm 2OG, 50 μm ammonium iron (II) sulfate. Concentrated stocks of 2OG, sodium l-ascorbate, and RPS23_47–84_ were prepared in 50 mm HEPES, pH 7.5; a 400 mm stock of ammonium iron (II) sulfate was prepared in 20 mm HCl, and dilutions were made in Milli-Q water. The reaction was initiated by addition of enzyme, incubated for 10 min at room temperature, and quenched with formic acid (1% final concentration). Hydroxylation of the RPS23 peptide was monitored by MALDI-TOF MS on a Bruker Autoflex Speed instrument in positive reflectron mode. Assay mixture (1 μl) was mixed with α-cyano-4-hydroxycinnamic acid matrix (1 μl; 1 mg/100 μl prepared in 50% acetonitrile) and applied onto a ground steel target.

## Author contributions

S. J. A., D. E. E., C. J. S., and P. J. O. conceptualization; S. J. A., D. E. E., C. J. S., and P. J. O. formal analysis; S. J. A., D. E. E., M. R. B., and P. J. O. validation; S. J. A., D. E. E., M. R. B., E. M. B., C. T. L., and P. J. O. investigation; S. J. A. and P. J. O. writing-original draft; S. J. A., D. E. E., M. R. B., E. M. B., C. T. L., C. J. S., and P. J. O. writing-review and editing; C. T. L., C. J. S., and P. J. O. funding acquisition; C. J. S. and P. J. O. supervision; C. J. S. and P. J. O. project administration.

## Supplementary Material

Supporting Information

## Footnotes

4 The abbreviations used are:

BER: base excision repair
DRR: direct reversal repair
ϵA: 1,*N*^6^-ethenoadenine
1mA: *N*^1^-methyladenine
3mA: *N*^3^-methyladenine
ϵC: 3,*N*^4^-ethenocytosine
7mG: *N*^7^-methylguanine
3mC: *N*^3^-methylcytosine
MMS: methyl methanesulfonate
2OG: 2-oxoglutarate
TCEP: tris(2-carboxyethyl)phosphine
ss: single-stranded
ds: double-stranded
MUG: mismatch-specific uracil-DNA glycosylase
YPD: yeast extract/peptone/dextrose
AAG: alkyladenine DNA glycosylase.
