# Supplementary material for: Expansion of base excision repair compensates for a lack of DNA repair by oxidative dealkylation in budding yeast
Source: J Biol Chem. 2019 Jul 18;294(37):13629–37. doi: 10.1074/jbc.RA119.009813 (PMC6746446; doi:10.1074/jbc.RA119.009813)
Supplement: Supporting Information [file supp_294_37_13629__index.html]

Expansion of base excision repair compensates for a lack of DNA repair by oxidative dealkylation in budding yeast — Expansion of base excision repair — Expansion of base excision repair compensates for a lack of DNA repair by oxidative dealkylation in budding yeast — Expansion of base excision repair — Supporting Information 

# Expansion of base excision repair compensates for a lack of DNA repair by oxidative dealkylation in budding yeast

## Supporting Information

- Supporting Information (to be published online) - 5 Tables and 10 Figures that provide experimental details and supporting experiments necessary for scientific rigor.
