# Supplementary material for: Expansion of base excision repair compensates for a lack of DNA repair by oxidative dealkylation in budding yeast
Source: J Biol Chem. 2019 Jul 18;294(37):13629–37. doi: 10.1074/jbc.RA119.009813 (PMC6746446; doi:10.1074/jbc.RA119.009813)
Supplement: Supporting Information [file supp_RA119.009813_153557_1_supp_365160_pffrmy.pdf]

Supporting Information

Expansion of base excision repair compensates for a lack of DNA repair by oxidative dealkylation

Suzanne J. Admiraal[1], Daniel E. Eyler[1#], Michael R. Baldwin[1], Emily M. Brines[1], Christopher T. Lohans[2], Christopher J. Schofield[2], and Patrick J. O'Brien[1]*

From the [1]Department of Biological Chemistry, University of Michigan Medical School, Ann Arbor, MI 48109-0600, USA; [2]Department of Chemistry, University of Oxford, Oxford, OX1 3TA, United Kingdom

*To whom correspondence should be addressed: Patrick J. O'Brien, Department of Biological Chemistry, University of Michigan Medical School, Ann Arbor, MI 48109-0600; pjobrien@umich.edu; Tel. (734) 647-5821; Fax. (734) 763-4581.

Table S1. Oligonucleotides tested as substrates.
Table S2. Domain analysis of selected proteins in the Fe(II)/2OG-dependent dioxygenase superfamily.
Table S3. *S. cerevisiae* strains.
Table S4. Oligonucleotides used for yeast strain construction and verification.
Table S5. Expected amplicon sizes for yeast strain verification.
Figure S1. Single-turnover excision of alkylated nucleobases by Mag1.
Figure S2. Mag1 does not excise alkylated nucleobases from ssDNA oligonucleotides.
Figure S3. Linear initial rates for Mag1-catalyzed glycosylase activity in substrate competition reactions.
Figure S4. Alkylated DNA is repaired by AlkB but not by Tpa1.
Figure S5. Domain analysis of selected proteins in the Fe(II)/2OG-dependent dioxygenase superfamily.
Figure S6. Single-turnover excision of alkylated nucleobases by AlkA.
Figure S7. Linear initial rates for AlkA-catalyzed glycosylase activity in substrate competition reactions.
Figure S8. Verification of URA3 integration in yeast strains.
Figure S9. Verification of *mag1Δ::kanMX*, *tpa1Δ::kanMX*, and *mag1Δ::LEU2 tpa1Δ::kanMX* strains.
Figure S10. SDS-PAGE gel of purified proteins.

Table S1. Oligonucleotides tested as substrates.

**Duplex DNA[a]**	**Sequence**
εA-25mer	5'-(FAM)-CGATAGCATCCT**εA**CCTTCTCTCCAT<br>3'-GCTATCGTAGGA T GGAAGAGAGGTA
1mA-25mer	5'-(FAM)-CGATAGCATCCT**1mA**CCTTCTCTCCAT<br>3'-GCTATCGTAGGA T GGAAGAGAGGTA
εC-25mer	5'-(FAM)-CGATAGCATCCT**εC**CCTTCTCTCCAT<br>3'-GCTATCGTAGGA G GGAAGAGAGGTA
3mC-25mer	5'-(FAM)-CGATAGCATCCT**3mC**CCTTCTCTCCAT<br>3'-GCTATCGTAGGA G GGAAGAGAGGTA
εA-19mer	5'-(FAM)-TAGCATCCT**εA**CCTTCTCTC<br>3'-ATCGTAGGA T GGAAGAGAG

[a]Abbreviations: FAM, 5'-fluorescein (6-FAM); εA, 1,N^6-ethenoadenine; 1mA, N^1-methyladenine; εC, 3,N^4-ethenocytosine; 3mC, N^3-methylcytosine.

Table S2. Conserved domain analysis of selected proteins in the Fe(II)/2OG-dependent dioxygenase superfamily, including Tpa1 and AlkB, generated using the Conserved Domain Database from the NCBI (https://www.ncbi.nlm.nih.gov/Structure/cdd/cdd.shtml). Results are depicted schematically in Figure S5.

Protein	Species	UniProt Entry	Amino Acids	Domain Name	Domain Accession	Domain Interval	E-value
Tpa1	*S. cerevisiae*	P40032	644	Ofd1_CTDD	pfam10637	333-638	3.47E-122
				EGL9	COG3751	9-262	5.02E-96
				P4Hc	smart00702	51-246	5.03E-34
				2OG-FeII_Oxy_4	pfam13661	145-246	3.64E-28
				2OG-FeII_Oxy_3	pfam13640	145-246	1.22E-17
OGFOD1	*H. sapiens*	Q8N543	542	2OG-FeII_Oxy_4	pfam13661	141-238	6.30E-40
				Ofd1_CTDD	pfam10637	261-542	1.08E-33
				P4Hc	smart00702	72-238	1.31E-21
				EGL9	COG3751	68-240	3.49E-18
				2OG-FeII_Oxy_3	pfam13640	141-238	3.50E-18
Sud1	*D. melanogaster*	Q9I7H9	536	Ofd1_CTDD	pfam10637	300-536	1.84E-66
				2OG-FeII_Oxy_4	pfam13661	172-274	3.26E-26
				P4Hc	smart00702	88-274	7.89E-25
				EGL9	COG3751	103-278	5.52E-22
				2OG-FeII_Oxy_3	pfam13640	171-274	6.08E-19
Ofd1	*S. pombe*	Q11120	515	Ofd1_CTDD	pfam10637	279-515	3.22E-113
				EGL9	COG3751	3-241	8.24E-97
				2OG-FeII_Oxy_4	pfam13661	128-229	1.31E-40
				P4Hc	smart00702	34-229	7.74E-29
				2OG-FeII_Oxy_3	pfam13640	128-229	4.15E-17
AlkBH1	*H. sapiens*	Q13686	389	alkb	TIGR00568	119-289	1.36E-93
				2OG-FeII_Oxy_2	pfam13532	98-299	5.13E-40
				AlkB	COG3145	160-294	5.23E-22
				PRK15401	PRK15401	170-299	3.44E-18
AlkBH2	*H. sapiens*	Q6NS38	261	2OG-FeII_Oxy_2	pfam13532	72-254	8.75E-31
				AlkB	COG3145	62-251	1.57E-29
				PRK15401	PRK15401	157-255	2.79E-05
AlkBH3	*H. sapiens*	Q96Q83	286	2OG-FeII_Oxy_2	pfam13532	90-275	4.70E-28
				AlkB	COG3145	82-272	5.35E-22
AlkB	*D. melanogaster*	Q7KUZ2	332	alkb	TIGR00568	103-262	6.17E-49
				2OG-FeII_Oxy_2	pfam13532	75-267	4.55E-45
				PRK15401	PRK15401	164-262	3.00E-23
				AlkB	COG3145	164-264	1.77E-21
Abh1	*S. pombe*	O60066	302	alkb	TIGR00568	65-263	2.91E-91
				AlkB	COG3145	54-265	1.55E-51
				2OG-FeII_Oxy_2	pfam13532	61-296	3.32E-45
				PRK15401	PRK15401	190-298	1.04E-15
AlkB	*E. coli*	P05050	216	PRK15401	PRK15401	1-212	1.35E-162
				alkb	TIGR00568	21-189	2.02E-104
				2OG-FeII_Oxy_2	pfam13532	18-210	4.75E-84
				AlkB	COG3145	1-207	1.04E-81

Table S3. *S. cerevisiae* strains.

**Strain**	**Parental Strain**	**Genotype**
MAG1 TPA1 (yDE54)	BY4741	*URA3 leu2Δ0 his3Δ1 met15Δ0*
mag1Δ (yDE58)	BY4741	*URA3 leu2Δ0 his3Δ1 met15Δ0 mag1Δ::kanMX*
tpa1Δ (yDE62)	BY4741	*URA3 leu2Δ0 his3Δ1 met15Δ0 tpa1Δ::kanMX*
mag1Δ tpa1Δ (yDE254)	yDE62	*URA3 leu2Δ0 his3Δ1 met15Δ0 tpa1Δ::kanMX mag1Δ::LEU2*

Table S4. Oligonucleotides used for yeast strain construction and verification.[a]

Oligo name	Sequence
tpa1_A2	CAAGCTGATGCAATGAGAGATCC
tpa1_B	TCGGTCTTAGAACCAGACAATTTAC
tpa1_C	CTAGCTGTATTTCACAAGAGCACAA
tpa1_D2	AAAGCCATTAGACGGTGCCAGGC
mag1_A2	CGGCCTGCAGTAATGCTATTAGATCAG
mag1_B	TAGATCTTCAGGAGCAAATACATCC
mag1_C	GAAAAAGAATTAATGCGTGAAAGAA
mag1_D2	GCGATGACCTCTTTGCATGTTATGATGG
kan_B	CTGCAGCGAGGAGCCGTAAT
kan_C	TGATTTTGATGACGAGCGTAAT
leu_B	TTTGATTCTGTGCGATAGCG
leu_C	GTTCCAACAGTACCACCGAAGT
ura3_A2	GCGAGGCATATTTATGGTGAAGGATAAG
ura3_B	TGGGACCTAATGCTTCAACTAA
ura3_C	GGGTGGAAGAGATGAAGGTTAC
ura3_D2	CCTGTCTTATTGTTCTTGATTTGTGCCCCG
MAG1::*LEU2* upstream	GGGATTGGTCTCAATGCCTGATTCAAGAAATATCTTGACC
MAG1::*LEU2* downstream	CCTAACTTAAAGATATCATGTGCGTATATAGTTTCGTCTACCC
ura3Δ0::*URA3* upstream	GGTTAATGTGGCTGTGGTTTC
ura3Δ0::*URA3* downstream	GTTCTGGCGAGGTATTGGATAG

[a] Flanking verification primers (A2, D2) were located farther upstream and downstream of the locus of interest than the primers used by the deletion project consortium and are thus annotated “A2” and “D2” rather than “A” and “D”.

Table S5. Expected amplicon sizes for yeast strain verification.

**forward primer(s)**	**reverse primer(s)**	**expected amplicon size in strain (nt)**			
		MAG1 TPA1 **(yDE54)**	***mag1Δ*** **(yDE58)**	***tpa1Δ*** **(yDE62)**	***mag1Δ tpa1Δ*** **(yDE254)**
mag1_A2	mag1_B<br>kan_B<br>leuB	1015	686	1015	532
mag1_C<br>kan_C<br>leu_C	mag1_D2	712	1126	712	685
mag1_A2	mag1_D2	1775	2470	1775	2550
tpa1_A2	tpa1_B<br>kan_B	795	795	665	665
tpa1_C<br>kan_C	tpa1_D2	863	863	975	975
tpa1_A2	tpa1_D2	2649	2649	2298	2298
ura3_A2	ura3_B	458	458	458	458
ura3_C	ura3_D2	471	471	471	471
ura3A2	ura3_D2	1333	1333	1333	1333

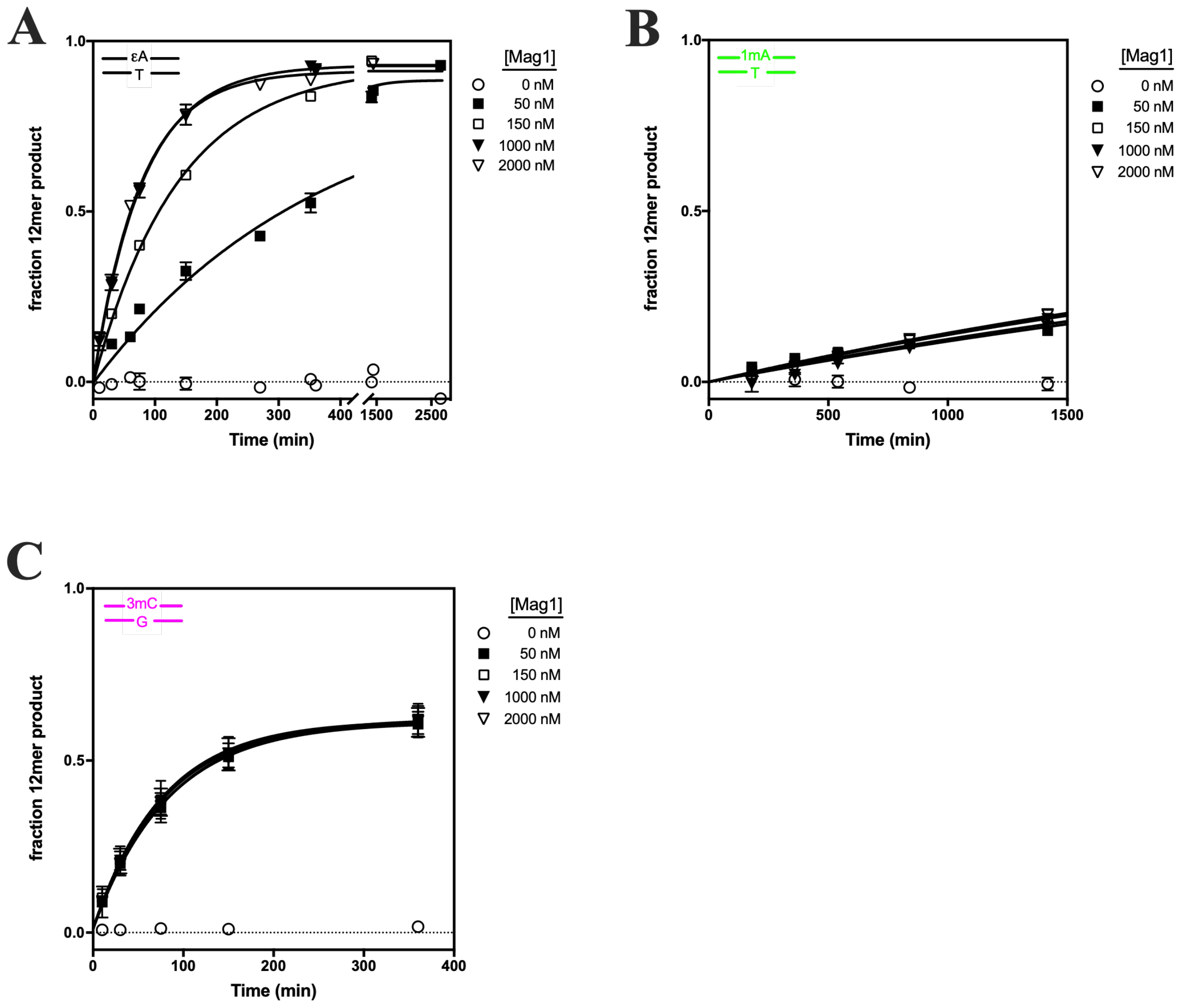

Figure S1. Single-turnover excision of alkylated nucleobases from 5 nM εA-25mer (A), 1mA-25mer (B), and 3mC-25mer (C) with varying concentrations of Mag1. The data were fit by a single exponential. The slower reactions of 1mA-25mer with Mag1 were not followed to completion, so an endpoint of 0.60 was used for this substrate because this is its endpoint after reacting to completion with *B. subtilis* AAG (data not shown). The average of duplicate reactions is shown ± SD.

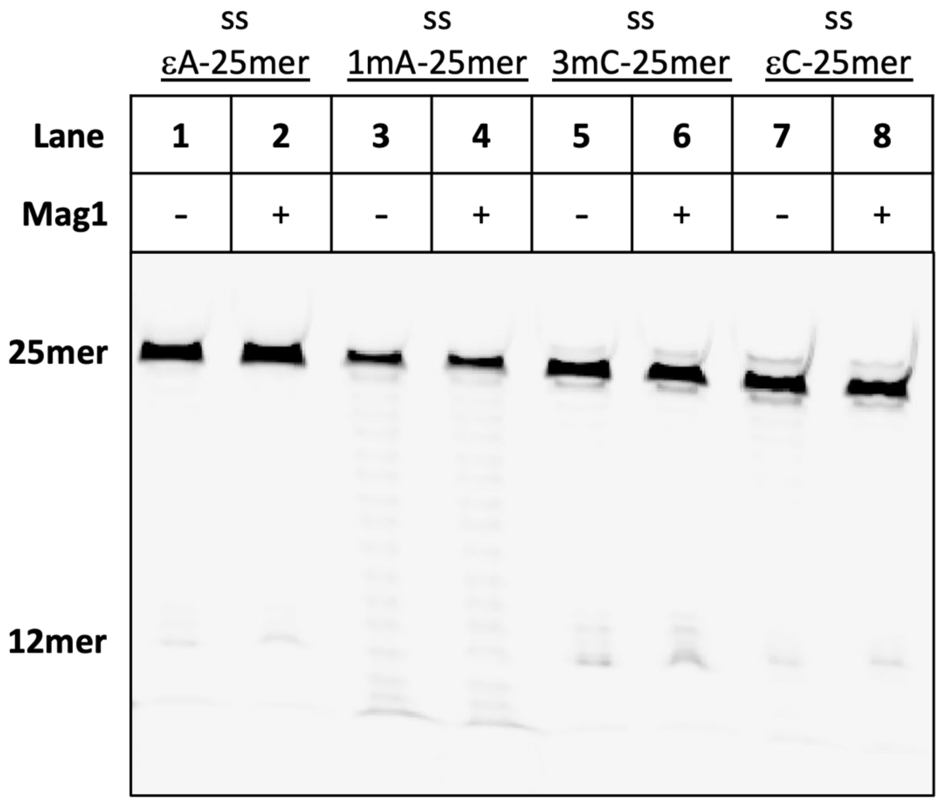

Figure S2. Mag1 does not excise alkylated nucleobases from ssDNA oligonucleotides. After 24 hr incubation of ssDNA oligonucleotides (25 base pairs) containing the indicated central alkylated base with or without Mag1, samples were treated with sodium hydroxide to cleave any abasic sites that were present and then analyzed on a 17.5% polyacrylamide gel under denaturing conditions.

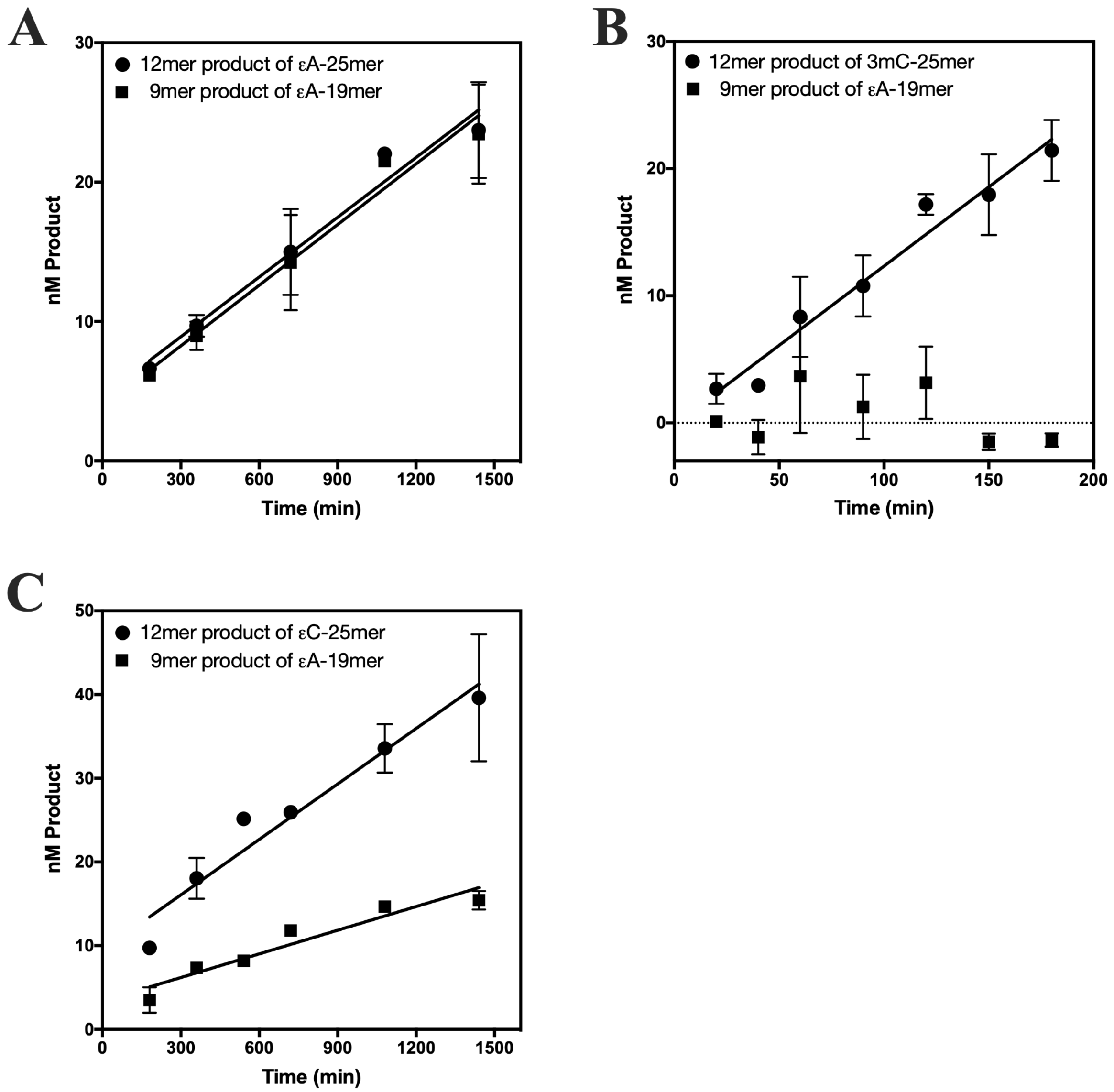

Figure S3. Linear initial rates for Mag1-catalyzed glycosylase activity toward mixtures of the reference substrate, εA-19mer (Table S1), and εA-25mer (A), 3mC-25mer (B), or εC-25mer (C) substrates. Cleavage of the reference substrate gives a labeled 9mer product and cleavage of each 25mer gives a labeled 12mer product. (A) The relative k_{cat}/K_M value of 0.98 ± 0.13 for εA-25mer with respect to εA-19mer was determined from the linear initial rates and the initial substrate concentrations (see Experimental Procedures). Mag1 was 50 nM, and εA-25mer and εA-19mer were 500 nM. (B) Reaction of εA-19mer could not be detected in the presence of 3mC-25mer, so a limit of >60 for the relative k_{cat}/K_M value of 3mC-25mer was obtained from the observed initial rate of formation for the 12mer product, an initial rate of formation of 0.01 nM/min (the detection limit for these reactions) for the 9mer product of εA-19mer, and the initial substrate concentrations (see Experimental Procedures). Mag1 was 50 nM, 3mC-25mer was 300 nM, and εA-19mer was 1500 nM. (C) The relative k_{cat}/K_M value of 2.3 ± 0.5 for εC-25mer with respect to εA-19mer was determined from the linear initial rates and the initial substrate concentrations (see Experimental Procedures). Mag1 was 50 nM, and εC-25mer and εA-19mer were 500 nM.

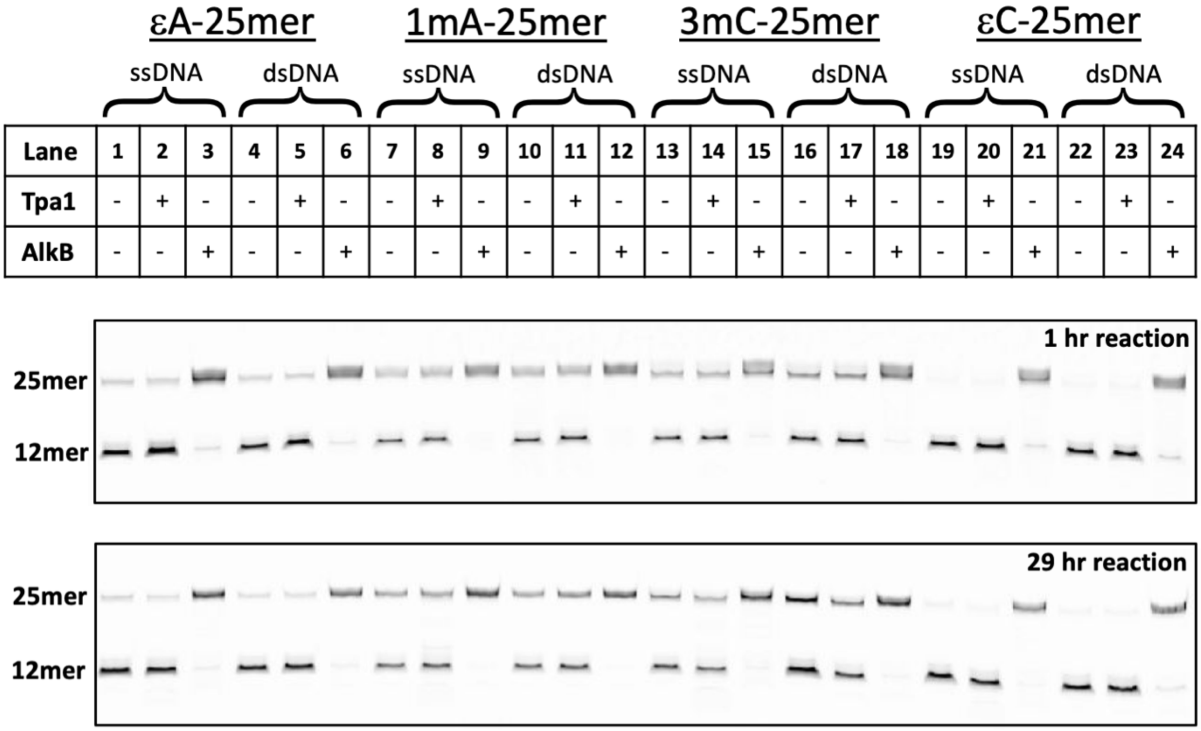

Figure S4. AlkB repairs both ssDNA and dsDNA 25mer oligonucleotides containing a central εA, 1mA, 3mC, or εC, but no DNA repair activity is observed for Tpa1. Each DNA substrate (100 nM) was incubated with no protein, 5 μM Tpa1, or 5 uM AlkB at 37 °C for 1 hr (upper gel) or 29 hr (lower gel). Quenched reactions were then treated with an appropriate glycosylase and hydrolyzed to convert any unrepaired 25mer DNA into 12mer DNA, as described in the Experimental Procedures. Treated samples were analyzed on 20% polyacrylamide gels under denaturing conditions.

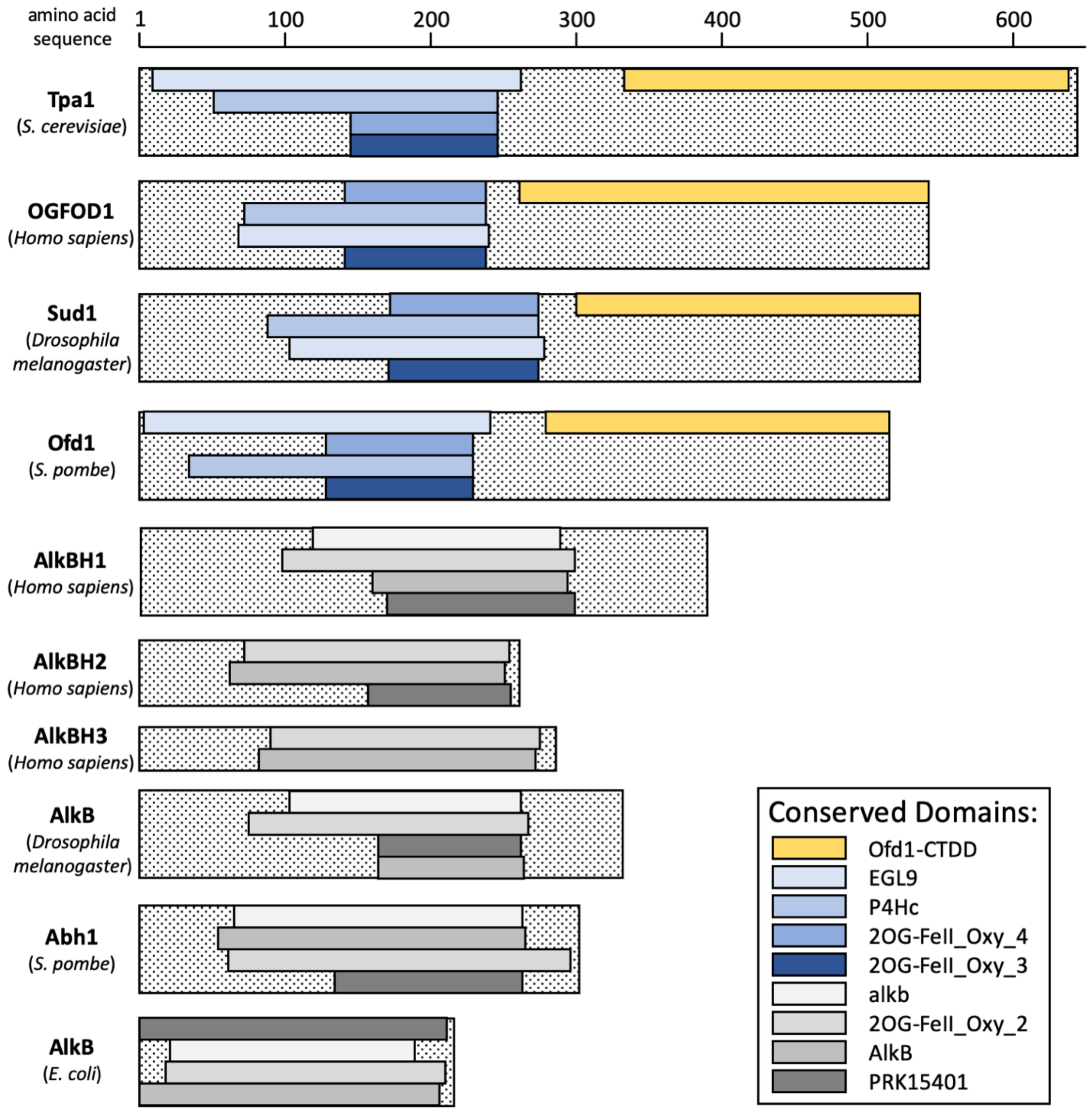

Figure S5. Conserved domain analysis of selected proteins in the Fe(II)/2OG-dependent dioxygenase superfamily, including Tpa1 and AlkB, generated using the Conserved Domain Database from the NCBI (https://www.ncbi.nlm.nih.gov/Structure/cdd/cdd.shtml). All conserved domains that were identified using an E-value threshold of 0.01 in full results mode are depicted above and summarized in Table S2.

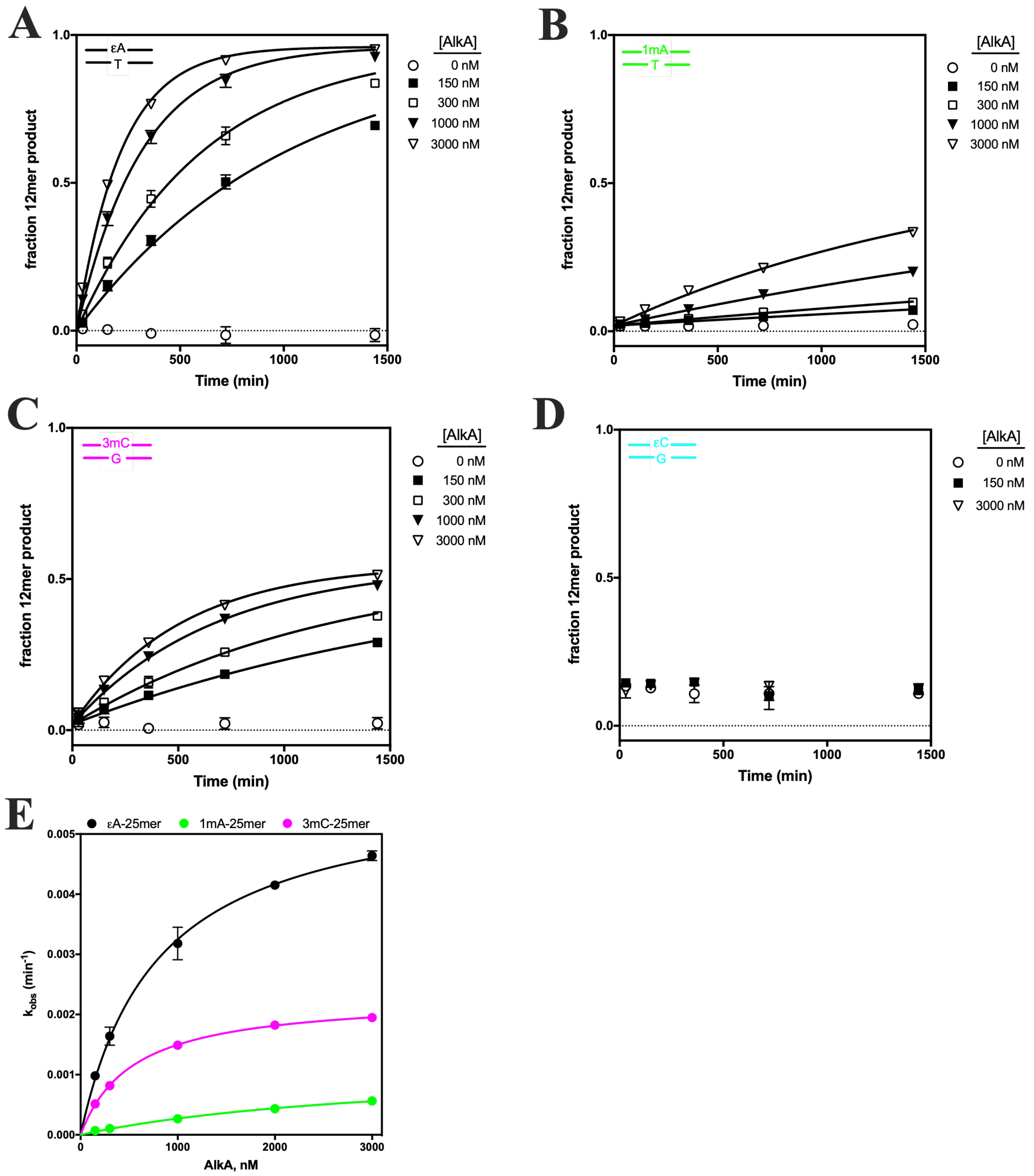

Figure S6. Single-turnover excision of alkylated nucleobases from 5 nM εA-25mer (A), 1mA-25mer (B), 3mC-25mer (C), and εC-25mer (D) with varying concentrations of AlkA. No reaction was observed for εC-25mer (D), so we calculated a limit of $k_{max}/K_{1/2}$ <12 $M^{-1}min^{-1}$ for εC-25mer as follows: if present, 5% product could readily be detected by the 1440 min timepoint in εC-25mer reactions containing 3000 nM AlkA (D, ▽), which corresponds to a k_{obs} of 0.05/1440 min = 3.5×10^{-5} min^{-1} and a $k_{max}/K_{1/2} = 3.5\times10^{-5}$ $min^{-1}/3\times10^{-6}$ M = 12 $M^{-1}min^{-1}$. Excision curves for the remaining substrates were fit to a single exponential. Reactions of 1mA-25mer (B) with AlkA were not followed to completion, so an endpoint of 0.60 was used for this substrate because this is its endpoint after reacting to completion with *B. subtilis*

AAG (data not shown). The average of duplicate reactions is shown ± SD. (E) Dependence of the single-turnover rate constant for alkylated base excision from εA-25mer, 1mA-25mer, and 3mC-25mer substrates on the AlkA concentration. Hyperbolic dependence of the single-turnover rate constant on the AlkA concentration was observed, with k_{max} of 0.0058 ± 0.0001 min^{-1} and a $K_{1/2}$ value of 780 ± 40 nM for εA-25mer, k_{max} of 0.0012 ± 0.0001 min^{-1} and a $K_{1/2}$ value of 3,400 ± 600 nM for 1mA-25mer, and k_{max} of 0.0023 ± 0.0001 min^{-1} and a $K_{1/2}$ value of 540 ± 10 nM for 3mC-25mer. The average of duplicate reactions is shown ± SD.

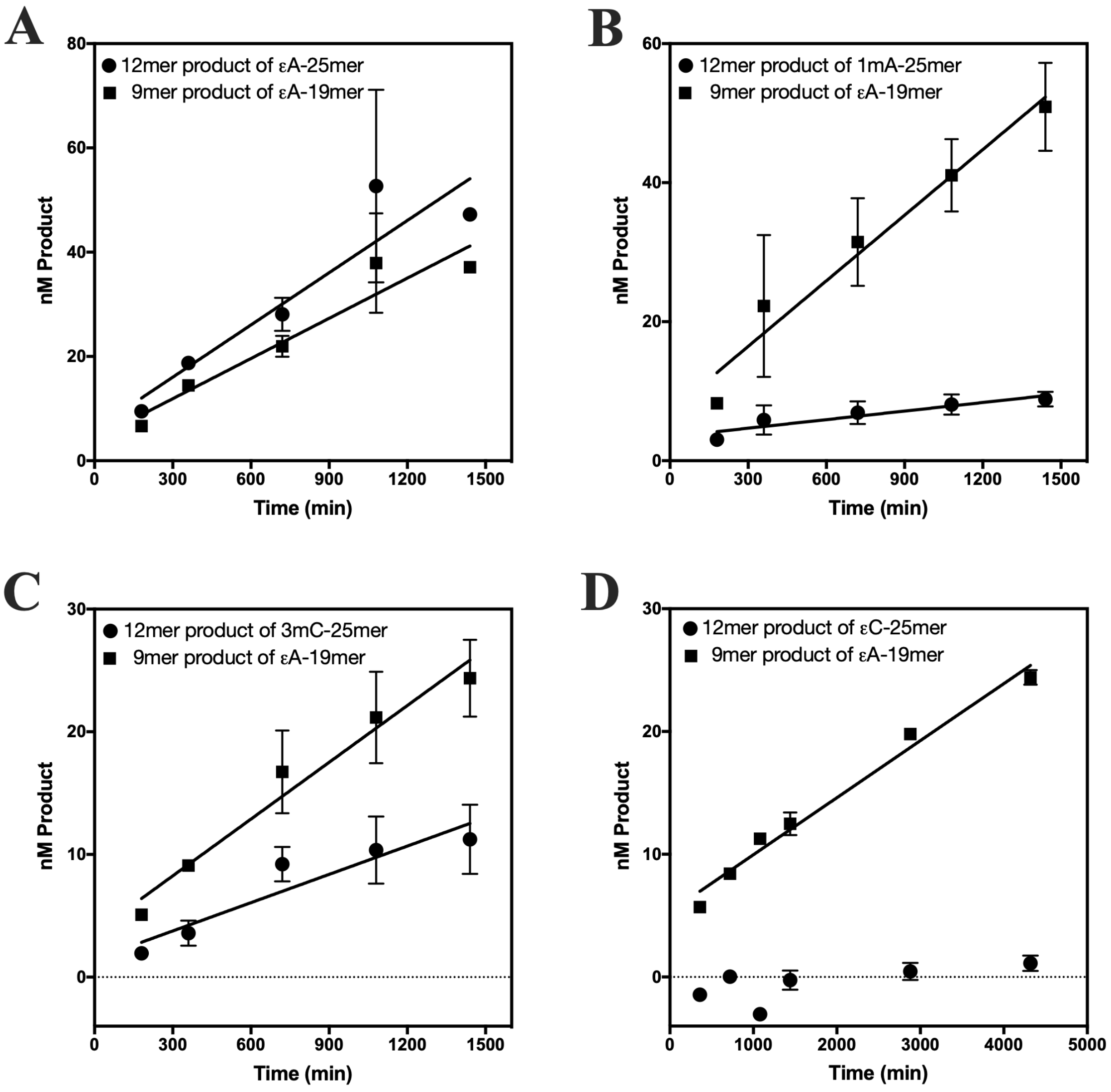

Figure S7. Linear initial rates for AlkA-catalyzed glycosylase activity toward mixtures of the reference substrate, εA-19mer (Table S1), and εA-25mer (A), 1mA-25mer (B), 3mC-25mer (C), or εC-25mer (D) substrates. Cleavage of the reference substrate gives a labeled 9mer product and cleavage of each 25mer gives a labeled 12mer product. (A) The relative k_{cat}/K_M value of 1.3 ± 0.4 for εA-25mer with respect to εA-19mer was determined from the linear initial rates and the initial substrate concentrations (see Experimental Procedures). Mag1 was 50 nM, and εA-25mer and εA-19mer were 500 nM. (B) The relative k_{cat}/K_M value of 0.21 ± 0.05 for 1mA-25mer with respect to εA-19mer was determined from the linear initial rates and the initial substrate concentrations (see Experimental Procedures). Mag1 was 50 nM, 1mA-25mer was 300 nM, and εA-19mer was 500 nM. (C) The relative k_{cat}/K_M value of 0.79 ± 0.18 for 3mC-25mer with respect to εA-19mer was determined from the linear initial rates and the initial substrate concentrations (see Experimental Procedures). Mag1 was 50 nM, 3mC-25mer was 300 nM, and εA-19mer was 500 nM. (D) Reaction of εC-25mer could not be detected in the presence of εA-19mer, so a limit of <0.1 for the relative k_{cat}/K_M value of εC-25mer was obtained from the observed initial rate of formation of 0.005 nM/min for the 9mer product, an initial rate of formation of 0.0005 nM/min (the detection limit for these reactions) for the 12mer product of εC-25mer, and the initial substrate concentrations (see Experimental Procedures). Mag1 was 50 nM, and εC-25mer and εA-19mer were 500 nM.

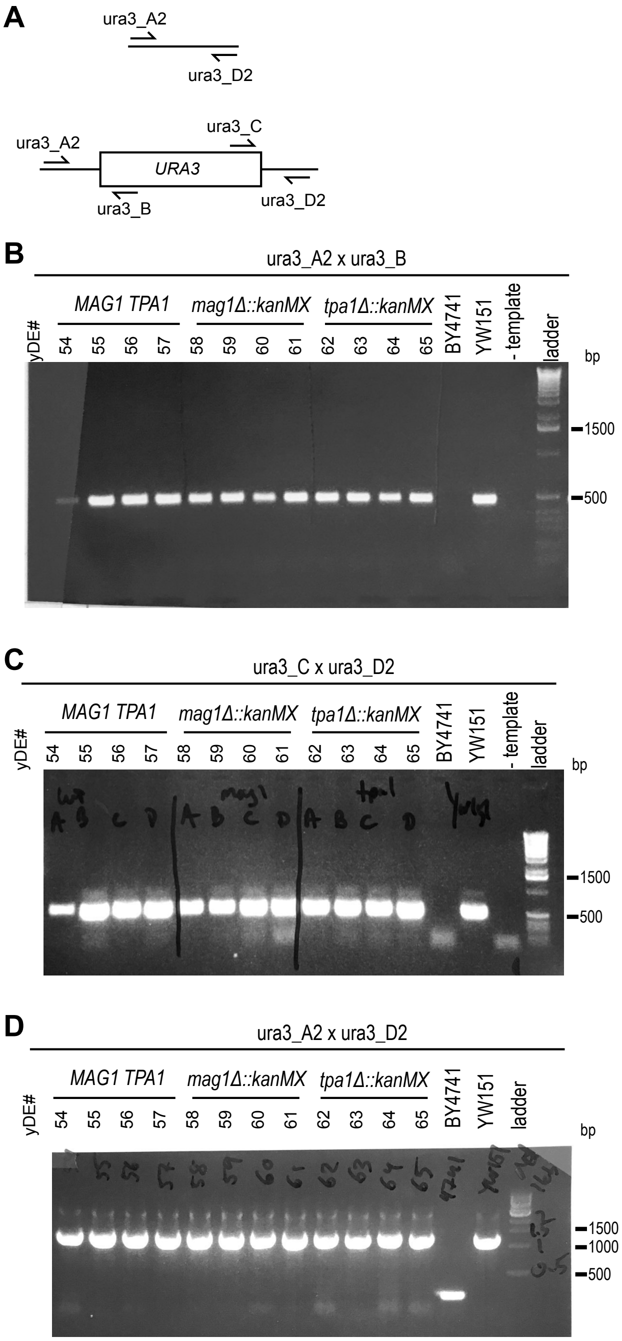

Figure S8. Verification of URA3 integration into BY4741, *mag1Δ::kanMX*, and *tpa1Δ::kanMX* yeast strains. The *URA3* locus was amplified from YW151 by PCR and transformed into the BY4741, *mag1Δ::kanMX*, and *tpa1Δ::kanMX* strains. Four clones were tested for each strain; subsequent experiments used strains yDE54 (*URA3 MAG1 TPA1*), yDE58 (*URA3 mag1Δ::kanMX*), and yDE62 (*URA3 tpa1Δ::kanMX*). Expected amplicon sizes are in Table S5. (A) Schematic showing the relative positions of the PCR primers in the BY4741 strain and in the *URA3* locus in the YW151 strain, which was used as the source of the wild-type *URA3* locus. (B) Agarose gel of PCR reactions using primer pair ura3_A2 and ura3_B to report on the 5' junction of the integration site. (C) Agarose gel of PCR reactions using primer pair ura3_C and ura3_D2 to report on the 3' junction of the integration site. (D) Agarose gel of PCR reactions using the primer pair ura3_A2 and ura3_D2 to report on the entire *URA3* locus.

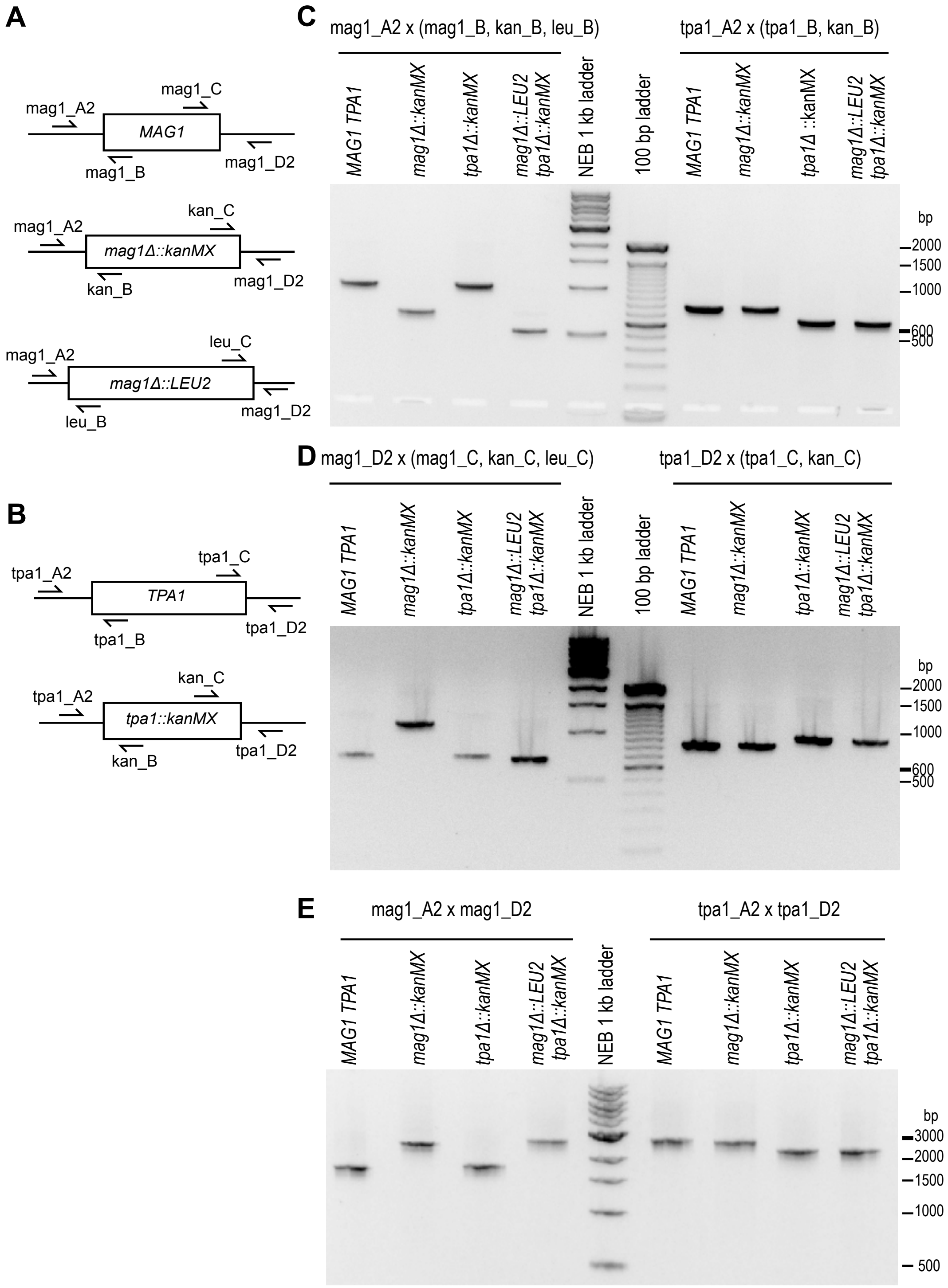

Figure S9. Verification of *mag1Δ::kanMX*, *tpa1Δ::kanMX*, and *mag1Δ::LEU2 tpa1Δ::kanMX* yeast strains. Expected amplicon sizes are in Table S5. (A) Schematic showing the relative locations of the PCR primers used to verify integrations at the *MAG1* locus. (B) Schematic showing the relative positions of the PCR primers used to verify integrations at the *TPA1* locus. (C) Agarose gel of PCR reactions targeting the 5' junctions at the *MAG1* and *TPA1* loci. (D) Agarose gel of PCR reactions targeting the 3' junctions of the *MAG1* and *TPA1* loci. (E) Agarose gel of PCR reactions amplifying the entire *MAG1* and *TPA1* loci.

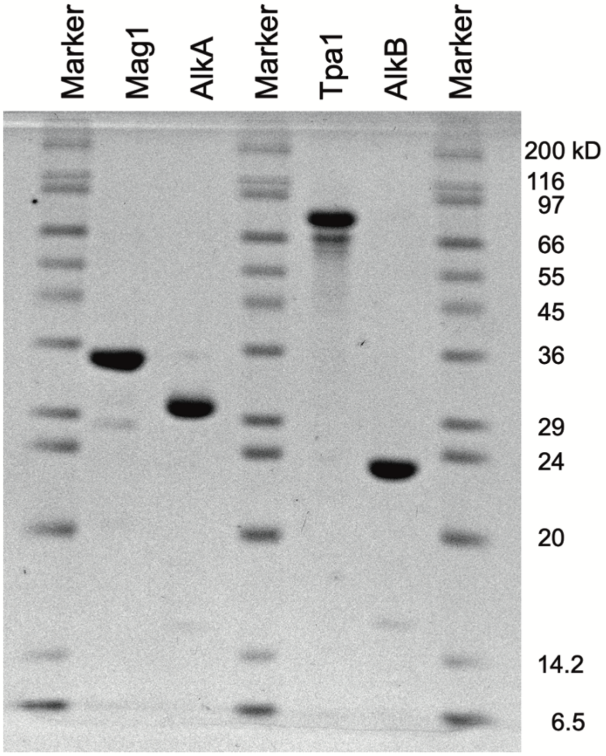

Figure S10. SDS-PAGE gel of purified proteins used in this study: Mag1 (35 kD), AlkA (31 kD), Tpa1 (74 kD), and AlkB (24 kD).
